# Tracking the Corticospinal Tract in Patients With High-Grade Glioma: Clinical Evaluation of Multi-Level Fiber Tracking and Comparison to Conventional Deterministic Approaches

**DOI:** 10.3389/fonc.2021.761169

**Published:** 2021-12-14

**Authors:** Andrey Zhylka, Nico Sollmann, Florian Kofler, Ahmed Radwan, Alberto De Luca, Jens Gempt, Benedikt Wiestler, Bjoern Menze, Sandro M. Krieg, Claus Zimmer, Jan S. Kirschke, Stefan Sunaert, Alexander Leemans, Josien P. W. Pluim

**Affiliations:** ^1^ Biomedical Engineering, Eindhoven University of Technology, Eindhoven, Netherlands; ^2^ Department of Diagnostic and Interventional Radiology, University Hospital Ulm, Ulm, Germany; ^3^ Department of Diagnostic and Interventional Neuroradiology, School of Medicine, Klinikum rechts der Isar, Technical University of Munich, Munich, Germany; ^4^ TUM-Neuroimaging Center, Klinikum rechts der Isar, Technical University of Munich, Munich, Germany; ^5^ Department of Radiology and Biomedical Imaging, University of California, San Francisco, San Francisco, CA, United States; ^6^ Image-Based Biomedical Modeling, Department of Informatics, Technical University of Munich, Munich, Germany; ^7^ TranslaTUM - Central Institute for Translational Cancer Research, Technical University of Munich, Munich, Germany; ^8^ Department of Imaging and Pathology, Translational MRI, Katholieke Universiteit (KU) Leuven, Leuven, Belgium; ^9^ Department of Neurosciences, Leuven Brain Institute (LBI), Katholieke Universiteit (KU) Leuven, Leuven, Belgium; ^10^ Image Sciences Institute, University Medical Center Utrecht, Utrecht, Netherlands; ^11^ Neurology Department, UMC Utrecht Brain Center, University Medical Center Utrecht, Utrecht, Netherlands; ^12^ Department of Neurosurgery, School of Medicine, Klinikum rechts der Isar, Technical University of Munich, Munich, Germany; ^13^ Department of Quantitative Biomedicine, University of Zurich (UZ), Zurich, Switzerland; ^14^ Department of Radiology, Universitair Ziekenhuis (UZ) Leuven, Leuven, Belgium

**Keywords:** fiber tractography, diffusion MRI, brain tumor, corticospinal tract (CST), neurosurgery planning

## Abstract

While the diagnosis of high-grade glioma (HGG) is still associated with a considerably poor prognosis, neurosurgical tumor resection provides an opportunity for prolonged survival and improved quality of life for affected patients. However, successful tumor resection is dependent on a proper surgical planning to avoid surgery-induced functional deficits whilst achieving a maximum extent of resection (EOR). With diffusion magnetic resonance imaging (MRI) providing insight into individual white matter neuroanatomy, the challenge remains to disentangle that information as correctly and as completely as possible. In particular, due to the lack of sensitivity and accuracy, the clinical value of widely used diffusion tensor imaging (DTI)-based tractography is increasingly questioned. We evaluated whether the recently developed multi-level fiber tracking (MLFT) technique can improve tractography of the corticospinal tract (CST) in patients with motor-eloquent HGGs. Forty patients with therapy-naïve HGGs (mean age: 62.6 ± 13.4 years, 57.5% males) and preoperative diffusion MRI [repetition time (TR)/echo time (TE): 5000/78 ms, voxel size: 2x2x2 mm^3^, one volume at b=0 s/mm^2^, 32 volumes at b=1000 s/mm^2^] underwent reconstruction of the CST of the tumor-affected and unaffected hemispheres using MLFT in addition to deterministic DTI-based and deterministic constrained spherical deconvolution (CSD)-based fiber tractography. The brain stem was used as a seeding region, with a motor cortex mask serving as a target region for MLFT and a region of interest (ROI) for the other two algorithms. Application of the MLFT method substantially improved bundle reconstruction, leading to CST bundles with higher radial extent compared to the two other algorithms (delineation of CST fanning with a wider range; median radial extent for tumor-affected vs. unaffected hemisphere – DTI: 19.46° vs. 18.99°, p=0.8931; CSD: 30.54° vs. 27.63°, p=0.0546; MLFT: 81.17° vs. 74.59°, p=0.0134). In addition, reconstructions by MLFT and CSD-based tractography nearly completely included respective bundles derived from DTI-based tractography, which was however favorable for MLFT compared to CSD-based tractography (median coverage of the DTI-based CST for affected vs. unaffected hemispheres – CSD: 68.16% vs. 77.59%, p=0.0075; MLFT: 93.09% vs. 95.49%; p=0.0046). Thus, a more complete picture of the CST in patients with motor-eloquent HGGs might be achieved based on routinely acquired diffusion MRI data using MLFT.

## Introduction

Gliomas represent the most common malignant brain tumors in adults, with an average annual age-adjusted incidence rate of ~4.67 to 5.73 per 100,000 population (1, 2). Anaplastic astrocytoma and glioblastoma are the major high-grade glioma (HGG) entities and peak in elderly subjects (1–3). Overall prognosis is poor, with a median survival below 2 years (1, 2, 4). HGGs can be regarded as chronic progressive diseases and typically show infiltrative growth behavior, which renders curative treatment almost impossible for the majority of affected patients (3, 5).

Nowadays, the standard treatment approach in patients harboring HGGs is a combination of neurosurgical resection, extended focal radiotherapy, and adjuvant chemotherapy (6–9). Nonetheless, multiple factors including histopathological characteristics, molecular tumor biology, as well as functional eloquence of the affected brain region contribute to individual therapy decision-making in clinical practice (9, 10). Regarding neurosurgical resection, a maximum extent of resection (EOR) has been associated with prolonged survival rates and better quality of life (11–17). However, mostly depending on individual tumor location, achieving a maximum EOR can be in conflict with preserving specific functions, such as the ability to move or speak without constraints. Thus, the principle of contemporary brain tumor surgery aims at an optimum EOR whilst avoiding surgery-related functional decline as far as possible (18, 19).

The gold-standard method for spatially resolved assessment of brain function is intraoperative direct electrical stimulation (DES), which can be applied as a strategy to guide neurosurgical resection and to avoid functional deficits in the course of tumor resection (8, 20–22). In addition to intraoperative DES, presurgical imaging is paramount to achieve an optimized onco-functional result. At the forefront of imaging techniques, multi-sequence magnetic resonance imaging (MRI) is applied to gather insights into spatial location, spread, and phenotyping of brain tumors (23–26). Lately, diffusion tensor imaging (DTI) in particular has seen increasing relevance as it allows identifying and delineating subcortical white matter (WM) structures non-invasively (27–31). In the neurosurgical context, DTI is frequently used for tracking of the corticospinal tract (CST), the main WM pathway subserving human motor function. The popularity of DTI can be explained by the low false-positive rate of tractography maps (32). Yet, the approach tends to produce underrepresented fiber bundles (33). Consequently, this causes an ongoing debate on whether conventional DTI methods are accurate and reliable enough to serve as a workable solution for delineating WM architecture in patients with glioma (34–37). Specifically, one main criticism is that the brain’s WM architecture harbors numerous fiber crossings and further complex geometrical configurations, including fiber branching, which are hard to resolve (38, 39).

While deterministic tractography with DTI is the most common preoperative approach, a variety of models has been proposed to overcome the overall limitations of DTI-based tractography and improve the reconstruction of WM fiber organization as an attempt to further narrow the gap between imaging and reality, including diffusion kurtosis imaging and fiber orientation distribution (FOD)-based approaches using constrained spherical deconvolution (CSD) as the most prominent representative (40–42). At the same time, although these models are more capable of disentangling fiber orientations, tractography algorithms commonly impose additional constraints that are set to achieve structurally plausible results, such as angular deviation and consideration of all the orientations as separate fibers. Consequently, either pathway propagation may be terminated, or a diverging branch may be pruned due to the angular limitations as well as related to spatial resolution constraints, which may artificially push the rate of false-negative findings (43). Thus, the possibility of incorporating fiber bifurcations with high angular deviations, such as those observed for the human CST, remains neglected. Probabilistic algorithms that are supposed to improve reconstructions by not just propagating into the peak FOD direction but sampling each step from the FOD could compensate for the angular resolution of the FOD model and capture certain pathway bifurcations. A series of previous publications has shown the ability of probabilistic tractography to improve the extent of the bundles over DTI-based tractography while highlighting microstructural changes induced by the tumor (44–46). However, usually direction samples are not drawn out of the whole FOD but from the segment defined by an angular deviation threshold (47). This introduces a limitation to probabilistic methods that is using higher angular deviation thresholds helps in reconstructing more complete bundles while also increasing the false-positive rate (32).

Against this background, we evaluate a novel method for improved fiber tractography of the CST in patients suffering from motor-eloquent HGGs, which aims to specifically tackle the issue of missing fiber branching of currently existing tractography procedures. We evaluate the recently developed multi-level fiber tracking (MLFT) approach that adds branches to the pathways that have been previously reconstructed, but do not reach a predefined target region (48, 49). Specifically, we hypothesize that the MLFT algorithm is capable of improving the reconstruction of the CST in the vicinity of a brain tumor when compared to conventionally used DTI-based tractography as well as tractography using CSD.

## Methods

### Study Design and Patient Inclusion

This study was approved by the local institutional review board and was conducted in accordance with the Declaration of Helsinki. The requirement for written informed consent was waived due to the study’s retrospective design.

Patients who underwent brain MRI using a multi-sequence imaging protocol for brain tumors according to clinical indication were retrospectively identified in the institutional Picture Archiving and Communication System (PACS). The time interval for PACS search ranged from February 2019 to February 2020 considering the time point of MRI acquisition. Inclusion criteria were 1) age above 18 years, 2) availability of preoperative 3-Tesla MRI including diffusion-weighted sequences, 3) diagnosis of a HGG (based on imaging findings and later confirmation by histopathological evaluation of biopsy probes or tumor tissue harvested during surgical resection), and 4) suspected motor-eloquent tumor location according to preoperative MRI (imaging suggesting infiltration or compression of anatomically suspected cortical motor-eloquent areas and/or suspected close proximity to the CST). The exclusion criteria were 1) artifacts due to implants or motion artifacts in imaging data according to visual image evaluation (e.g., non-diagnostic image quality due to patient movement during image acquisition), and 2) previous brain surgery.

Overall, 40 patients fulfilled the inclusion criteria and were considered for this study. Clinical details including demographics and final histopathological tumor grading were extracted from electronic health records of these patients.

### Magnetic Resonance Imaging

Cranial MRI was performed in the preoperative routine setting. All imaging considered in this study was acquired on the same two 3-Tesla scanners (Achieva dStream or Ingenia; Philips Healthcare, Best, Netherlands) using a 32-channel head coil.

The standardized multi-sequence imaging protocol for brain tumors included a three-dimensional (3D) fluid attenuated inversion recovery (FLAIR) sequence (repetition time [TR]/echo time [TE]: 4800/277 ms, 1 mm^3^ isovoxel covering the whole head), an axial T2-weighted sequence (TR/TE: 3396/87 ms, voxel size of 0.36×0.36×4 mm^3^), a diffusion-weighted sequence (TR/TE: 5000/78 ms, voxel size of 2x2x2 mm^3^, one volume at b = 0 s/mm^2^, 32 volumes at b = 1000 s/mm^2^), and a 3D T1-weighted turbo field echo (TFE) sequence (TR/TE: 9/4 ms, 1 mm^3^ isovoxel covering the whole head) without and with application of a contrast agent using a dose of 0.2 ml per kg body weight of gadoteric acid (Dotagraf 0.5 mmol/ml; Jenapharm GmbH & Co. KG, Jena, Germany). Further sequences not related to this study’s analyses were acquired by default and used for radiological reporting and image-based diagnostics.

### Data Processing

#### Co-Registration and Segmentation

First, to avoid errors in the automated structural parcellation due to the presence of pathology and related anatomical distortion, lesion filling for the T1-weighted images was done prior to structural parcellation, which substitutes the tumor volume in the image with data mimicking signal from the healthy tissue (either using noise or healthy tissue simulation). For robust parcellation, in this work we used automated Virtual Brain Grafting (VBG), which enables the generation of a virtual lesion-free T1-weighted image and structural parcellation using FreeSurfer recon-all (https://github.com/KUL-Radneuron/KUL_VBG/; KU Leuven, Department of Imaging and Pathology, Translational MRI, Leuven, Belgium) (50, 51). Lesion segmentation required for VBG was obtained fusing eight segmentation algorithms using majority voting from the Brain Tumor Segmentation (BraTS) toolkit (52, 53). The BraTS toolkit relies on a multi-modal input (non-contrast and contrast-enhanced T1-weighted images, FLAIR images, and T2-weighted images) and produces segmentation masks that enclose the tumor core (necrotic center and contrast-enhancing tumor parts) and FLAIR-hyperintense zones (edema/tumor infiltration), which were further used to compute the respective volumes (by accumulating volumes of each voxel in the respective masks) (52, 53). Before performing segmentations, all MRI data were transferred to Montreal Neurological Institute (MNI) space (with an isotropic voxel size of 1 mm^3^).

**Figure 1:**
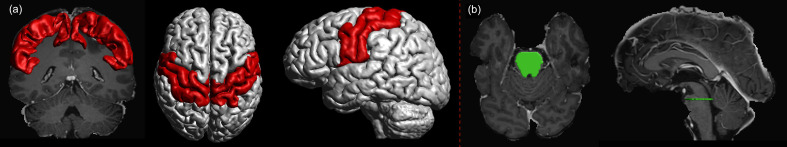
**(A)** Motor cortex mask (red) was assembled using precentral, postcentral, and paracentral gyri as segmented using FreeSurfer. The motor cortex mask was used as a target region. **(B)** The seed region (green) was defined as a cross-section of the brain stem at the pontine level.

The diffusion-weighted MRI data of the individual patients were corrected for motion and eddy currents, and co-registered to the corresponding T1-weighted images using *ExploreDTI* (version 4.8.6; http://www.exploredti.com/; PROVIDI Lab, Utrecht, Netherlands) (54). The FODs were estimated using recursive calibration of the response function (55). We used a spherical harmonics order of *L_max_* = 6. Motor cortex masks were assembled from precentral, postcentral, and paracentral lobule segmentations (**Figure 1A**) obtained with *FreeSurfer* (version 6.0.0; http://surfer.nmr.mgh.harvard.edu; Laboratory for Computational Neuroimaging, Charlestown, MA, USA) (51) using the Desikan-Killiany atlas (56). All image co-registrations and segmentations were visually inspected for quality and, in case of segmentations, manually corrected by a neuroradiologist when necessary.

#### Fiber Tacking Algorithms

Three deterministic tractography approaches were used in this study to reconstruct the CST of both hemispheres: DTI-based tractography, CSD-based tractography, and MLFT. DTI-based tractography was chosen since it is widely used in current clinical practice (36, 57). This algorithm propagates fiber streamlines into the main direction of the estimated diffusion tensor. However, it leads to issues with reconstructing pathways in certain complex situations (e.g., crossing or kissing fibers) when the estimated diffusion becomes closer to isotropic and the main tensor direction may not coincide with any of the underlying fiber orientations (38, 39). CSD-based tractography has improved the specificity compared to DTI-based tractography given higher angular resolution and an ability to disentangle more complex fiber configurations (41, 42). During pathway propagation each time the algorithm chooses an FOD peak that minimizes angular deviation from the previous step. The CSD approach was included as it has shown to be capable of adequately accounting for crossing fiber configurations, and it serves as a basis for the MLFT algorithm.

The recently proposed MLFT algorithm reconstructs bundles as multi-level structures, with the exact number of levels defined by the user (48, 49). Given a seed and a target region, MLFT aims to iteratively improve bundle reconstruction by adding pathways with high angulation reaching the target region (48, 49). At each iteration, MLFT propagates pathways from a set of seed points using deterministic CSD-based tractography that takes every step into the direction of a FOD peak the least deviating from the direction of the previous step. After propagation, the points of the pathways that did not reach the target region are used as seed points for the following iteration. Their initial directions are then defined as the peaks of the corresponding FODs that were ignored during propagation. If a seed point corresponds to multiple unused FOD peaks, it is duplicated to allow propagating each of the alternative directions. The rest of the pathways reaching the target region are forming a new level of the reconstruction. They are concatenated with the segment of the pathway they branched from that originates from the prior seed point set. This procedure is repeated for a predefined number of iterations. For the CST reconstruction, two levels (iterations) were used in this study. Thus, by extending the reconstruction with each new level, MLFT is attempting to account for branching fibers. It was also shown to preserve topography of the bundles (48, 49).

Additionally, it can be noticed that the reconstructions performed with deterministic CSD-based tractography are essentially the first level of the MLFT reconstructions. Thus, the extent of MLFT reconstructions will always at least cover that of the CSD-based algorithm.

#### Tractography Setup

To reconstruct the CST within each hemisphere, the seed region was placed in the single-slice transverse cross-section of the pontine level of the brain stem as obtained from brain parcellations (**Figure 1B**) (58). Motor cortex masks assembled from the segmentation of precentral, postcentral, and paracentral gyri of the left and right hemisphere, respectively, were set as target regions for MLFT and as a region of interest (ROI) to filter the results of DTI- and CSD-based tractography (**Figure 1A**). Five seed points were sampled per voxel in the seed mask at a single-slice level in the superior part of the brain stem, ensuring that all the points were on the same transverse plane. The tractography step was set to half a voxel size, the angular threshold was set to 45° (48, 49). For MLFT and CSD-based tractography the FOD peak threshold amplitude was set to 0.08, which was chosen empirically based on visual inspection of the results and with the aim to increase the volume of the reconstruction without introducing obvious false-positive pathways. For DTI-based tractography the fractional anisotropy (FA) threshold was set to 0.1. The number of iterations was set to 2 for MLFT. Additionally, due to reconstruction of CST branches for the left and right hemisphere from the same seed region in the brain stem, interhemispheric connections were filtered out. Additional experiments evaluating algorithms on fine-grained target regions are provided in the **Supplementary Material**.

### Fiber Tracking Evaluation

#### Qualitative Assessment

Visual image evaluation was performed by a neuroradiologist (7 years of experience in neuroradiological imaging) using *ExploreDTI*. The reconstructed bundles were rendered in the same scene as the contrast-enhanced T1-weighted images for an interactive assessment of the course of the CST and its relation to the tumor.

Patients were pseudonymized during all visual image evaluations. In detail, datasets stemming from DTI-based tractography, CSD-based tractography, and MLFT were opened during three rounds of evaluation, with each round randomly including one of those tractography results per patient. Between each round of assessment, an interval of at least two weeks was established to minimize recall bias. Both the tumor-affected and unaffected hemispheres were separately evaluated per patient. First, the course of the reconstructed CST through anatomical landmarks known to be key for the descending CST (ipsilateral internal capsule and cerebral crus at the level of the brain stem) was assessed in binary fashion (CST passing through/not passing through internal capsule and cerebral crus). Second, for tractography within the tumor-affected hemisphere, the neuroradiologist assessed whether the reconstructed CST appeared to be unaffected (no contact and no visually identifiable deviation), spatially deviated, infiltrated or split, or destroyed (entire or partial disintegration of the CST) due to the tumor mass, similar to previous work on qualitative evaluation of fiber tract anatomy (59).

#### Quantitative Assessment

Quantitative assessment of the CST bundles reconstructed with DTI-based, CSD-based tractography, and MLFT was performed, including radial extent and coverage of reconstructed fibers. The radial extent (in °) of the CST was calculated to show how much of the motor cortex is covered, which was achieved by computing the size of the segment of the coronal motor mask projection covered by the CST. Thus, the motor mask projection defines an arc of 90°, and the overlap of the bundle visitation mask on the motor cortex defines segments on the arc that constitute the radial extent. The difference in radial extents of the bundles reconstructed in tumor-affected and unaffected hemispheres was compared. Outliers in the difference distribution were detected as patients falling into the distribution tails and accounting for about 5% of the distribution. The threshold for the detection was calculated using the 2σ rule, where σ is the standard deviation (SD).

Given its wide use in clinical routine, DTI-based tractography was considered a baseline of comparison for the assessment of the algorithms regarding bundle trajectory. Thus, in order to assess the coherence of the CSD-based tractography and MLFT reconstructions with the DTI-based tractography results, coverage of the bundles generated with DTI-based tractography by the ones generated with CSD and MLFT was calculated. For calculation, binary visitation masks were created of the reconstructed bundles (with voxels being set to 1 if at least one pathway passed through it). Then, the part of the DTI-reconstructed bundle’s mask intersecting with corresponding masks of the MLFT and the CSD-based reconstructions was calculated (in %, where 90% DTI coverage by MLFT would mean that 90% of the CST volume reconstructed by DTI-based tractography is also included in the respective reconstructed bundle when MLFT is used as the tractography algorithm in the same patient). The masks consisted of the voxels visited by the corresponding bundle (voxel contains at least one pathway point). All computations for quantitative image assessment were performed using in-house developed MATLAB scripts (version R2018b; The MathWorks Inc., Natick, MA, USA).

### Statistics

For statistical data analyses, SPSS (version 26.0; IBM SPSS Statistics for Windows, IBM Corp., Armonk, NY, USA) and SciPy library [version 1.3.1; https://www.scipy.org/scipylib/ (60)] were used. In all statistical tests a significance level of α = 0.05 was used.

Descriptive statistics included mean ± SD, ranges, and absolute or relative frequencies. For qualitative image assessment in the tumor-affected hemisphere, Chi-squared tests were conducted to test for differences in the spatial characteristics of the CST (unaffected, spatially deviated, infiltrated/split, or destroyed) between DTI-based tractography, CSD-based tractography, and MLFT. For quantitative image assessment, the tractography algorithms were first compared to each other based on the radial extents of the reconstructions, separately for the unaffected and tumor-affected hemispheres and for the right versus left hemispheres, using two-sided Wilcoxon signed-rank tests. Furthermore, Wilcoxon signed-rank tests were used to compare the radial extents achieved by the same algorithm in affected and unaffected hemispheres, respectively.

Additionally, the coverage of the DTI-based reconstruction of the CST by CSD-based tractography and MLFT was compared for unaffected and tumor-affected hemispheres using two-sided Wilcoxon signed-rank tests. This allowed to assess if the presence of the tumor and related mass effects caused a significant change in the results of MLFT and CSD-based tractography compared to DTI-based tractography for tract coverage. In addition, correlations between the ratio of DTI-based reconstructions of the CST covered by CSD-based tractography or MLFT with the tumor core volumes or FLAIR-hyperintensity zone volumes were calculated using Pearson correlation coefficients.

## Results

### Patient Cohort

Forty patients (mean age: 62.6 ± 13.4 years, age range: 29.5 – 85.9 years, 17 females and 23 males) with a diagnosis of HGG and suspected motor-eloquent tumor location were included. **Table 1** provides further cohort details.

**Table 1:** Characteristics of the study cohort.

Item		Value
**Age** (years; mean ± SD & range)		62.6 ± 13.4 (29.5 – 85.9)
**Sex** (% of patients)	Male	57.5
	Female	42.5
**Affected hemisphere** (% of patients)	Left	40.0
	Right	60.0
**Surgical procedure performed** (% of patients)	Biopsy	22.5
	Resection	67.5
	Resection & intraoperative RTX	10.0
**Extent of resection** (% of patients)	STR	25.8
	GTR	74.2
**Tumor grade** (% of patients)	WHO grade III	12.5
	WHO grade IV	87.5
**Tumor core volume** (mm^3^, mean ± SD & range)		47,997.4 ± 39,098.9 (2,582 – 170,576)
**Volume of FLAIR-hyperintense zone** (mm^3^, mean ± SD & range)		64,727.3 ± 48,394.8 (4,625 – 184,127)

SD, standard deviation; WHO, World Health Organization; STR, subtotal resection; GTR, gross total resection; RTX, radiotherapy.

### Qualitative Assessment

**Figure 2:**
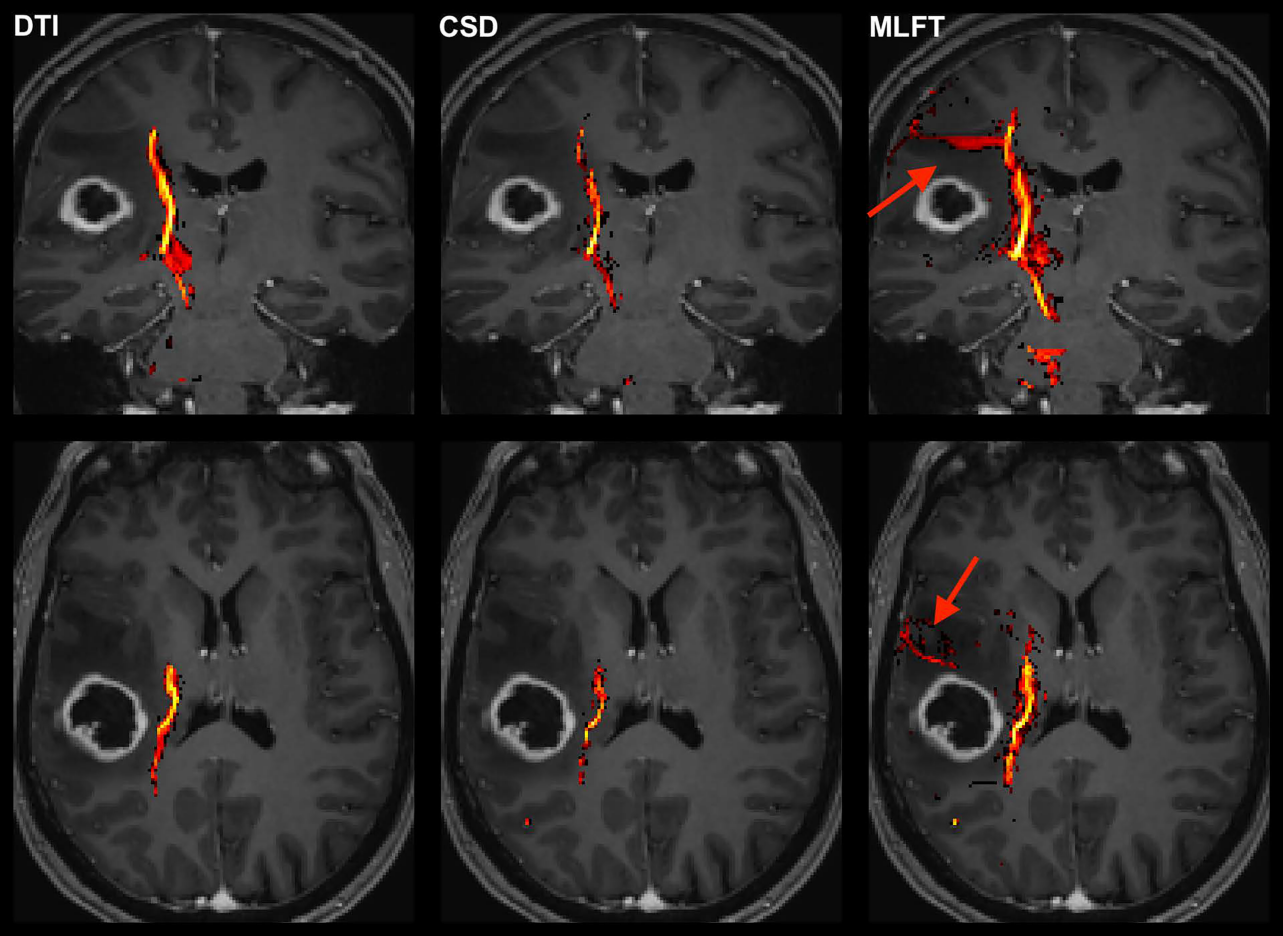
Exemplary case for reconstruction of the corticospinal tract (CST) depending on the algorithm used for tractography. This illustrative exemplary case of a patient suffering from a right-sided high-grade glioma (HGG) shows the reconstructed CST within the tumor-affected hemisphere as derived from diffusion tensor imaging (DTI)-based tractography, constrained spherical deconvolution (CSD)-based tractography, and multi-level fiber tracking (MLFT). The CST reconstructions are fused with axial and coronal contrast-enhanced T1-weighted images to outline the lesion-to-CST relationship as well as the CST volume and course. The MLFT approach enables fiber tracking with a larger radial extent, thus displaying also fanning of the CST and fibers with acute angles (red arrow).

**Figure 3:**
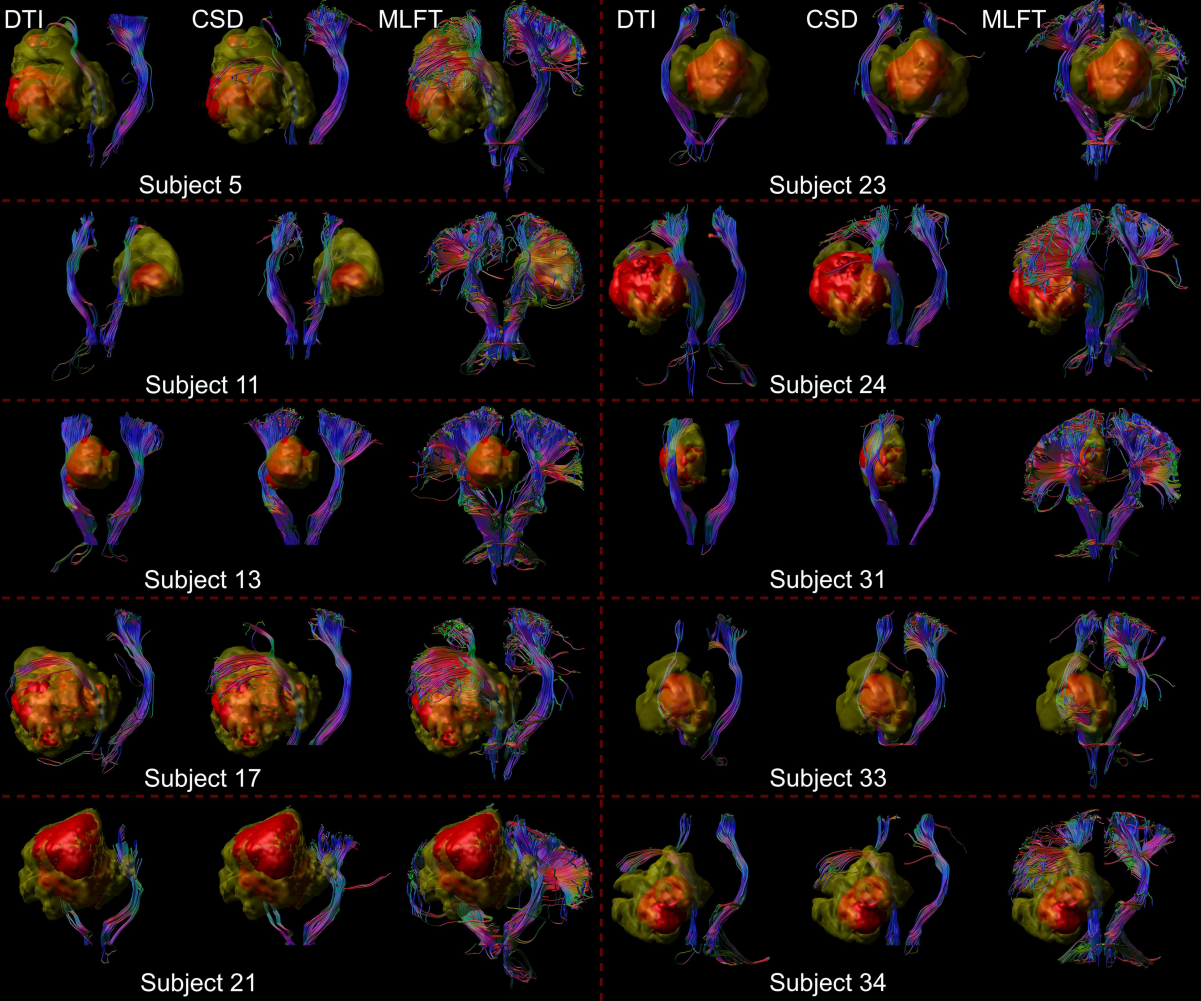
Comparison of reconstructions of the corticospinal tract (CST) depending on the algorithm chosen for tractography. This figure shows reconstructions of the CST within the tumor-affected and unaffected hemispheres in a subset of 10 patients from the cohort, using diffusion tensor imaging (DTI)-based tractography, constrained spherical deconvolution (CSD)-based tractography, and multi-level fiber tracking (MLFT). The tumor core is shown as a red volume, the hyperintense zone in fluid attenuated inversion recovery (FLAIR) sequences is shown as a yellow volume. While CSD-based tractography provides reconstructions comparable to DTI-based tractography, MLFT is able to improve depiction of the extent of the CST fanning of both tumor-affected and unaffected hemispheres.

Representative exemplary cases for CST reconstruction by DTI-based tractography, CSD-based tractography, and MLFT are shown in **Figures 2**, **3**. The reconstructed CST passed through the internal capsule and cerebral crus as key anatomical landmarks for both hemispheres of all enrolled patients.

The reconstructed CST bundle was unaffected by or not in contact with the tumor mass in n=15, 8, and 4 patients for DTI-based tractography, CSD-based tractography, and MLFT, respectively, and did not fulfill the criteria of a disintegrated course in any of the patients. Furthermore, the reconstructed CST bundle was deviated in n=24, 26, and 22 patients, respectively. It appeared to be infiltrated/split in n=1, 6, and 14 patients when using DTI-based tractography, CSD-based tractography, or MLFT, respectively. There was a statistically significant difference in these spatial characteristics of the CST depending on the tractography approach chosen (p = 0.0006).

### Quantitative Assessment

#### Radial Extent

**Figure 4:**
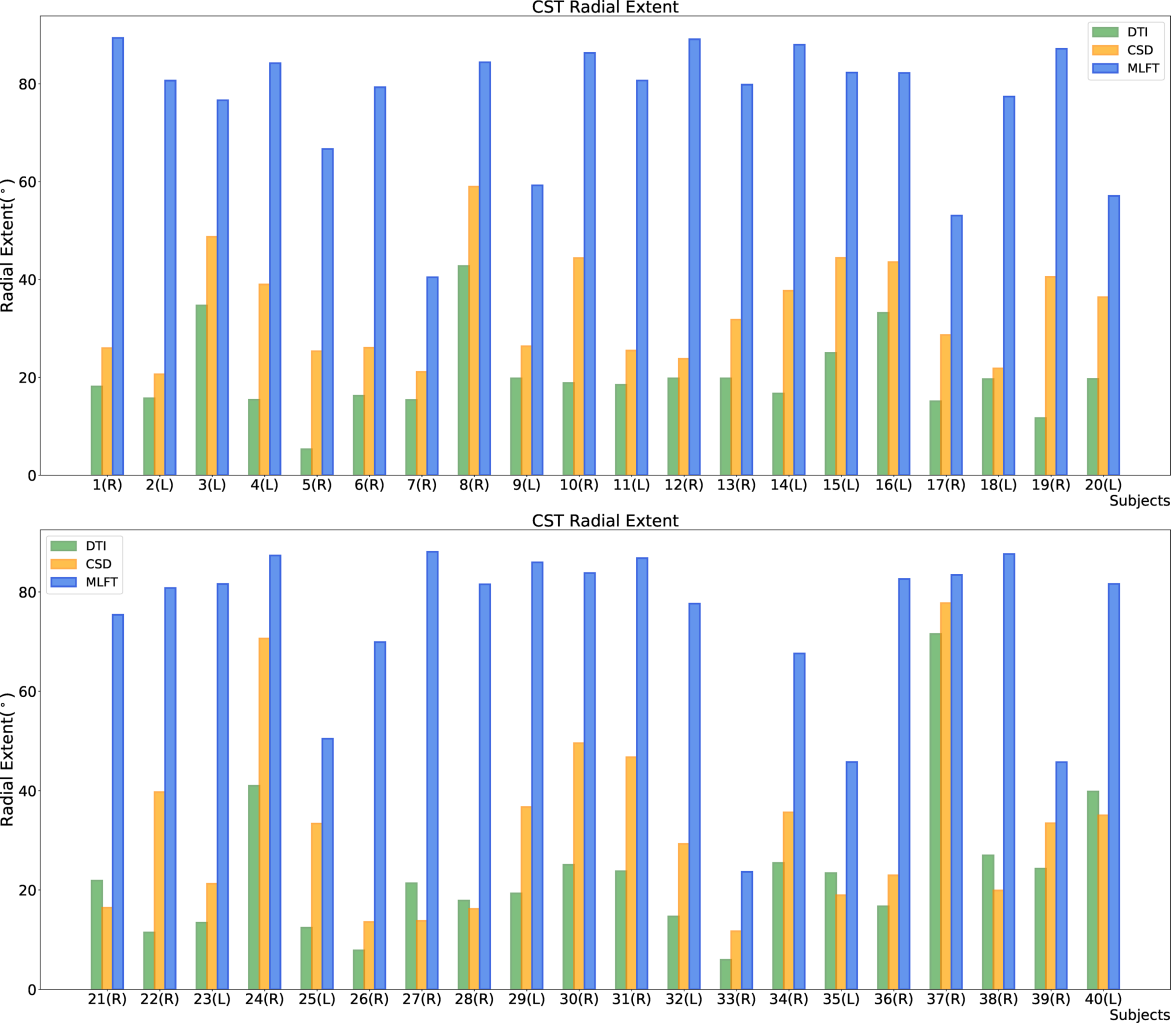
Comparison of the radial extent of the corticospinal tract (CST) branches of the tumor-affected hemispheres. This figure illustrates the radial extent for CST reconstruction using diffusion tensor imaging (DTI)-based tractography (green), constrained spherical deconvolution (CSD)-based tractography (orange), and multi-level fiber tracking (MLFT; blue). The hemisphere affected by the tumor per patient is indicated next to the subject index (L – left, R – right). Using MLFT led to CST reconstructions with larger radial extent in all patients.

**Figure 5:**
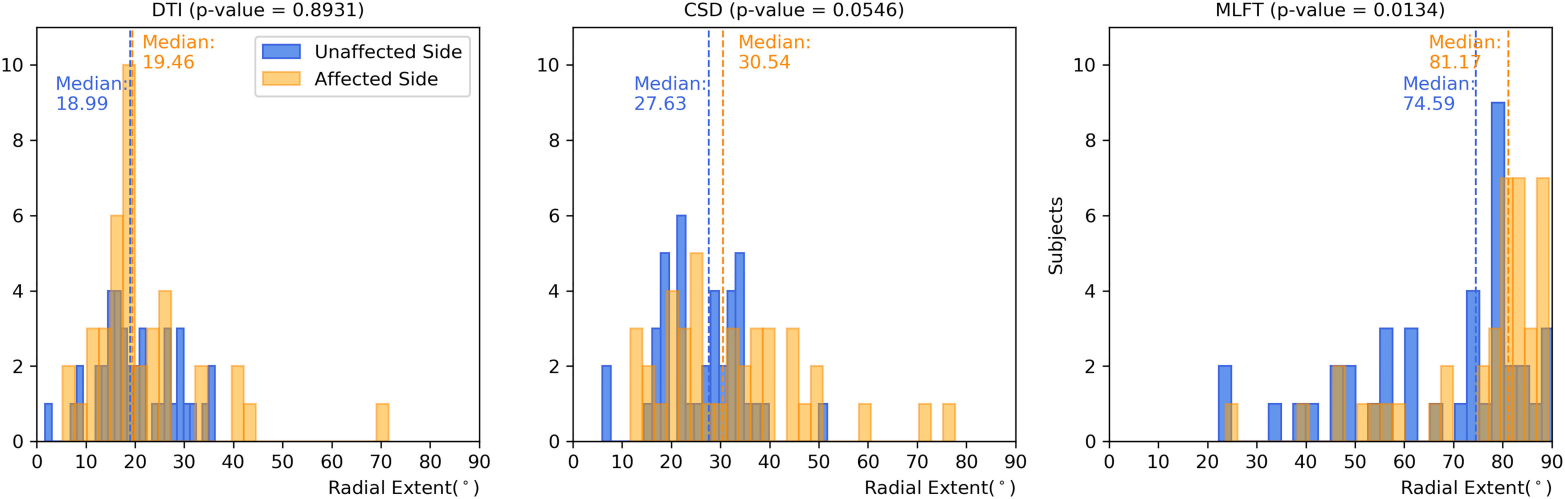
Comparison of the radial extent of the corticospinal tract (CST) branches. This figure shows the radial extents (with median values as vertical dashed lines) for the CST reconstructions derived from diffusion tensor imaging (DTI)-based tractography, constrained spherical deconvolution (CSD)-based tractography, and multi-level fiber tracking (MLFT). Columns for the tumor-affected hemispheres are displayed in orange, columns for the unaffected hemispheres are depicted in blue. The affected hemispheres show higher radial extent in case of each of the used tractography algorithms. The p-values are derived from comparisons between hemispheres per tractography algorithm (Wilcoxon signed-rank paired tests with significance level α=0.05).

The radial extents of the CST branches reconstructed with the three tractography algorithms are presented in **Figures 4**, **5**. The MLFT algorithm consistently provides increased radial extent when compared to both CSD-based and DTI-based tractography in all patients. In addition, when comparing radial extents between reconstructions from the three different tractography algorithms, results were statistically significant throughout (DTI vs. CSD/DTI vs. MLFT/CSD vs. MLFT: p < 0.05 each for tumor-affected vs. unaffected as well as right vs. left hemispheres; **Table 2**).

Each of the algorithms produced CST reconstructions with higher median radial extent of the reconstructions for the tumor-affected hemispheres as compared to the unaffected hemispheres, with a statistically significant difference only for MLFT (median radial extent for tumor-affected vs. unaffected hemisphere – DTI: 19.46° vs. 18.99°, p = 0.8931; CSD: 30.54° vs. 27.63°, p = 0.0546; MLFT: 81.17° vs. 74.59°, p = 0.0134).

**Figure 6:**
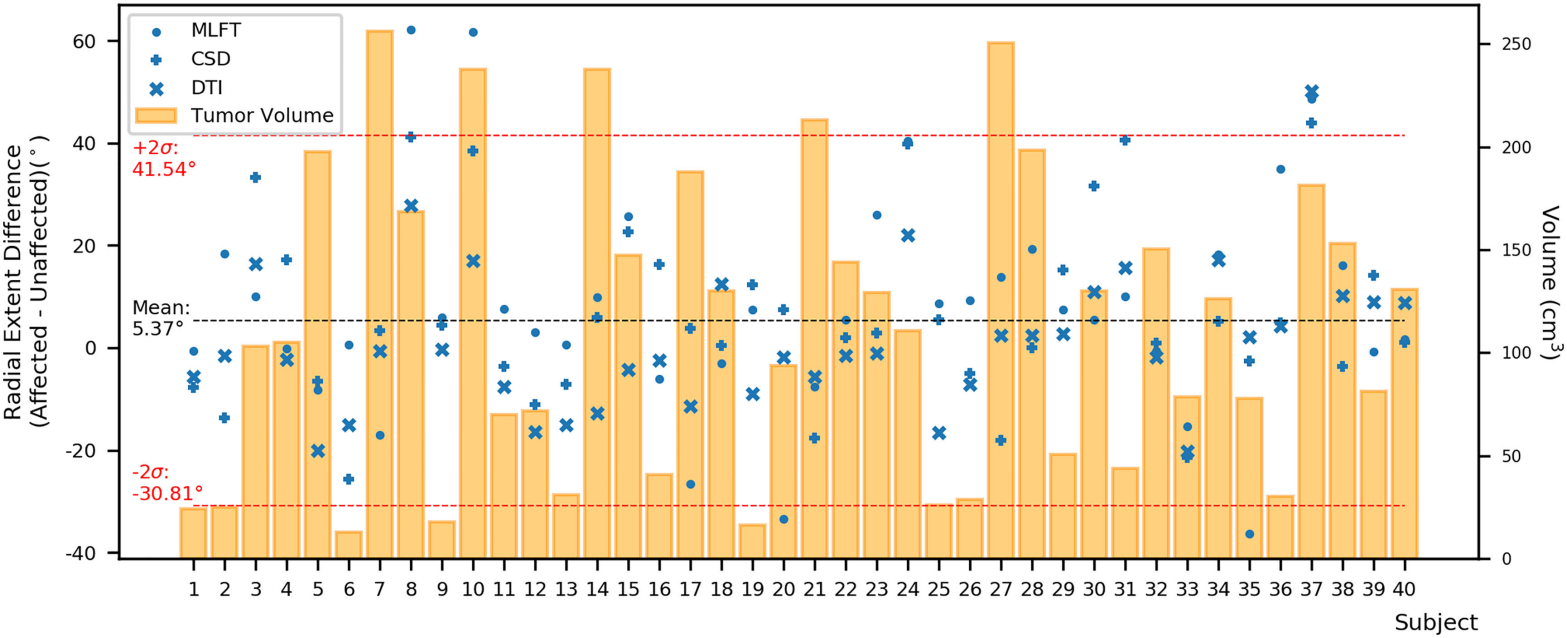
Differences between the radial extent of tumor-affected and unaffected hemispheres. This figure shows the radial extent differences in relation to combined tumor and FLAIR-hyperintense zone volumes (orange) using the mean (black dashed line) with +/- 2 standard deviation (SD, provided as σ; red dashed lines) to identify potential outliers. Circles represent data points for the corticospinal tract (CST) as derived from multi-level fiber tracking (MLFT), while + represents data points derived from constrained spherical deconvolution (CSD)-based tractography and x represents data points stemming from diffusion tensor imaging (DTI)-based tractography. The outliers with positive radial extent difference are of most interest as they show unexpected behavior with higher radial extent in the tumor-affected hemisphere.

**Figure 7:**
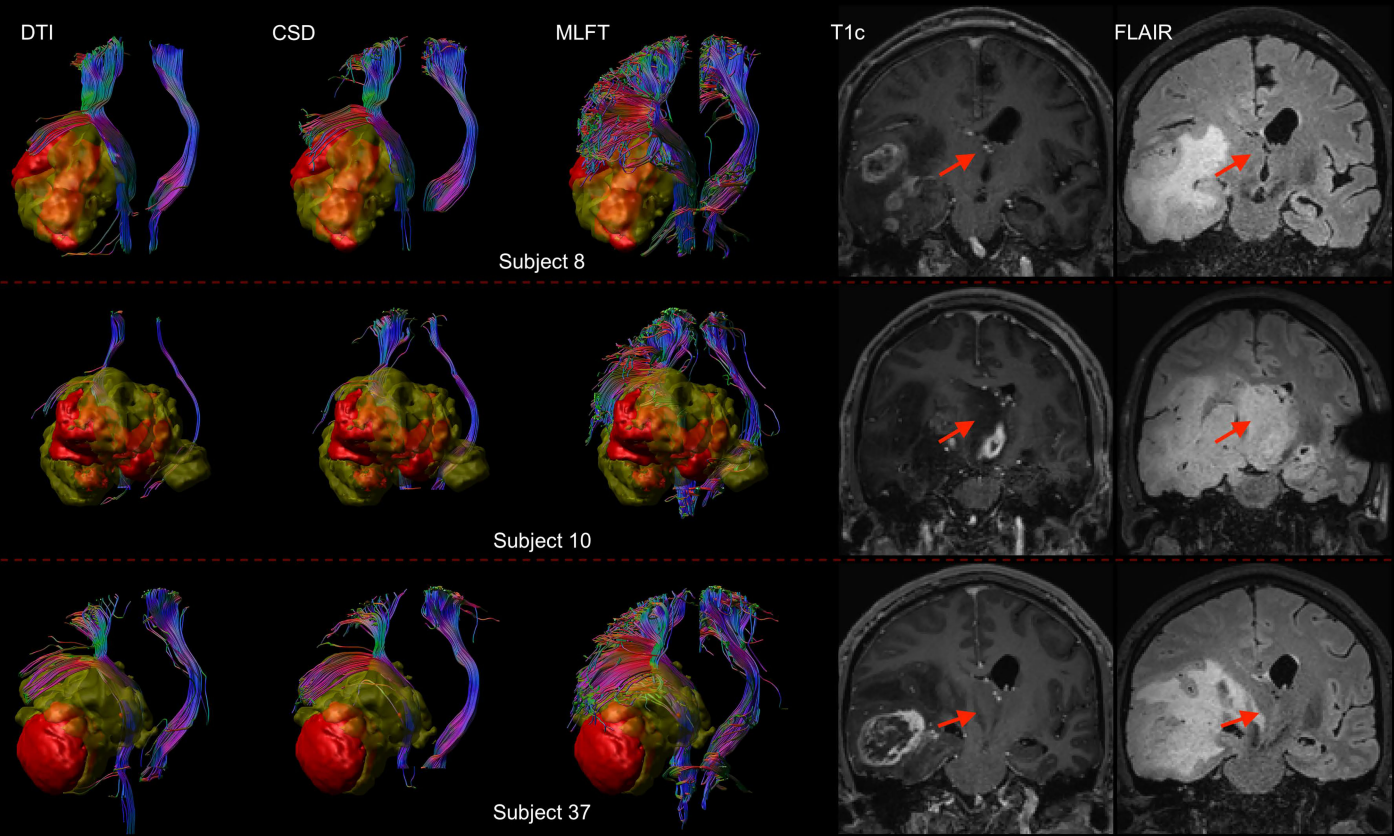
Comparison of reconstructions of the corticospinal tract (CST) depending on the algorithm chosen for tractography in patients with high radial extent in the tumor-affected hemisphere. This figure shows reconstructions of the CST using diffusion tensor imaging (DTI)-based tractography, constrained spherical deconvolution (CSD)-based tractography, and multi-level fiber tracking (MLFT) within the tumor-affected and unaffected hemispheres in the subset of the three patients that were identified as outliers regarding radial extent within affected hemispheres (considering a 2σ threshold). The tumor core is shown as a red volume, the hyperintense zone in fluid attenuated inversion recovery (FLAIR) sequences is shown as a yellow volume. These patients were all characterized by extensive mass effect that caused deformation of the CST bundle within the tumor-affected hemisphere as well as, to a lesser extent, within the unaffected hemisphere with considerable midline shift (red arrow in coronal contrast-enhanced T1-weighted and coronal FLAIR images). In all cases, fanning is considerably improved particularly in the tumor-affected hemispheres when using the MLFT algorithm, with only few fibers with acute angles being displayed adjacent to the tumor masses when using DTI-based tractography.

Furthermore, the differences in radial extents of the CST bundles in tumor-affected and unaffected hemispheres were compared (**Figure 6**). Using 2σ, three outliers were identified (patients #8, #10, and #37), who were all characterized by extensive mass effect that caused deformation of the CST bundle within the tumor-affected hemisphere, and to a lesser extent also a deviation of the CST within the unaffected hemisphere (**Figure 7**). Midline shifts can be observed in these three outliers.

#### Coverage of DTI

**Figure 8:**
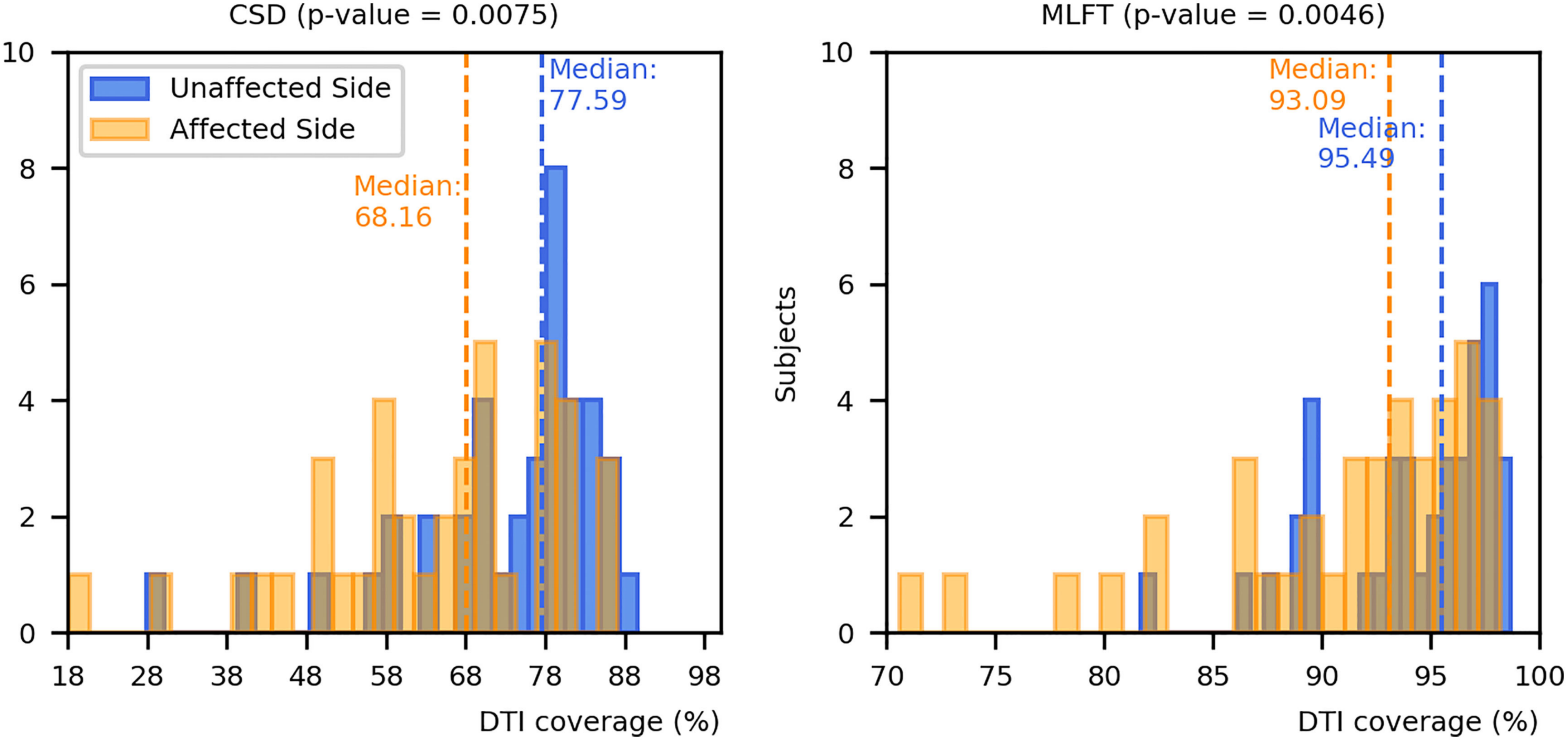
Comparison of coverage for corticospinal tract (CST) branches. This figure shows the coverage (with median values as vertical dashed lines) of the CST reconstructions derived from diffusion tensor imaging (DTI)-based tractography by constrained spherical deconvolution (CSD)-based tractography or multi-level fiber tracking (MLFT). Columns for the tumor-affected hemispheres are displayed in orange, columns for the unaffected hemispheres are depicted in blue. The affected hemispheres show lower coverage compared to the tumor-unaffected hemispheres. The p-values are derived from comparisons between hemispheres per tractography algorithm (Wilcoxon signed-rank paired tests with significance level α=0.05).

The results on comparing the coverage of DTI-based reconstructions by the corresponding CSD-based and MLFT-based reconstructions are depicted in **Figure 8**. MLFT provides a higher fraction of coverage of the DTI reconstruction results of the CST when compared to CSD.

Both CSD-based and MLFT results more clearly coincide with DTI-based reconstructions in case of the unaffected hemispheres (median coverage for affected vs. unaffected hemisphere – CSD: 68.16% vs. 77.59%, p = 0.0075; MLFT: 93.09% vs. 95.49%; p = 0.0046), while the reconstructions in the tumor-affected hemisphere are characterized by a higher mismatch. Yet, for the MLFT median coverage is >90% for both the unaffected and tumor-affected hemisphere, which contrasts with the CSD-based reconstruction with a median coverage of <80%. The two patients that had the lowest DTI coverage (lower than 75%) in the tumor-affected hemisphere by the MLFT reconstructions were subjects with extensive mass effects and high tumor volume, namely patients #5 and #17. The lowest DTI coverage achieved with CSD-based tractography is also attributed to patient #5. For these patients, tumor volumes as well as volumes of the FLAIR-hyperintense zones ranged in the upper quartile.

The correlation coefficients of the DTI coverage by CSD-based tractography or MLFT with the tumor core (necrotic center and contrast-enhancing tumor parts) and FLAIR-hyperintense zones are shown in **Table 3**. There were statistically significant negative correlations between the volume of the FLAIR-hyperintense zone and the DTI coverage (CSD: r = -0.52, p = 0.0006; MLFT: r = -0.52, p = 0.0005). Likewise, there were statistically significant negative correlations between the volume of the tumor core plus the volume of the FLAIR-hyperintense zone and DTI coverage (CSD: r = -0.48, p = 0.0018; MLFT: r = -0.47, p = 0.0021).

## Discussion

In this work we evaluated a novel tractography algorithm, MLFT, to achieve improved reconstruction of the CST in patients with motor-eloquent HGG. The MLFT algorithm was compared to deterministic DTI-based and deterministic CSD-based tractography of the CST of both hemispheres. The main findings are as follows: 1) the algorithm chosen for tractography had significant impact on the spatial course, volume, and shape of the CST, with MLFT-based reconstructions showing significantly higher radial extents; 2) compared to deterministic CSD-based tractography, MLFT showed higher coincidence with the DTI-based reconstructions, with a median coverage of >90% for both the tumor-affected as well as unaffected hemispheres; and 3) coverage of the CST as tracked by the DTI-based algorithm was significantly negatively associated with tumor-related mass effects (as estimated by volumes of tumor core and FLAIR-hyperintense zones) for both the CSD-based algorithm and MLFT.

Tractography of WM structures such as the CST is frequently employed for preoperative planning and intraoperative resection guidance in patients with intracranial neoplasms, using primarily DTI-based approaches (27–31). However, DTI-based tractography has several known limitations that may hamper value for clinical applications, including its limited ability to resolve geometrically complex situations such as crossing or kissing fibers (38, 39). Previous research has shown that using more advanced techniques, such as q-ball and CSD-based fiber tractography, may lead to improved results (37, 61, 62). In turn, the proposed MLFT algorithm is developed from CSD-based tractography and similarly propagates fiber pathways based on FOD peaks. However, unlike CSD-based tractography, MLFT assumes that FOD peaks represent not only fiber crossings, but also indicate fiber branching or high-angular deviation (48, 49). Without prior anatomical knowledge, such an approach would be at risk of generating multiple false-positive streamlines, which needs to be avoided particularly for ultimate clinical applicability. In order to prevent a high false-positive rate, MLFT requires well-defined target and seed regions, and if certain pathways do not enter the target area our algorithm checks if any deviation at the previous points would allow reaching the target region. Hence, using the algorithm gives some control over specificity while improving sensitivity.

Reconstructions of the CST using MLFT consistently showed the highest radial extent when compared to DTI- or CSD-based tractography (**Figure 3**). On average, CSD-based tractography achieved higher radial extent than the DTI-based algorithm (**Table 2**), while in some individual cases DTI-based tractography outperforms the CSD algorithm for this metric (**Figure 4**). Yet, MLFT provided CST branches with higher radial extents even for the tumor-affected hemispheres, which may indicate that a more complete reconstruction of particularly highly angulated parts of the CST close to its origin becomes possible when using MLFT (**Figures 4**, **5**). Indeed, based on simulations and preliminary *in-vivo* imaging in a cohort of healthy subjects, it has been suggested that the fanning close to the motor cortex can be well delineated with MLFT (48, 49). Considering the potential value of a broader fanning and reconstruction of laterally coursing fiber pathways, MLFT-derived reconstructions may be of merit since especially marginal fibers can be at risk for damage when aiming at a maximized EOR during surgery of motor-eloquent HGGs.

**Table 2:** Radial extent of fiber reconstructions.

Radial Extent Hemisphere	Mean ± SD, (°)			Range, (°)			P-value		
	DTI	CSD	MLFT	DTI	CSD	MLFT	CSD–DTI	MLFT–DTI	MLFT–CSD
**Right**	21.9 ± 11.7	30.0 ±14.6	73.8 ± 16.1	5.28 – 71.53	11.68 – 77.75	23.65 – 90.45	5.5*10^-5^	3.6*10^-8^	3.6*10^-8^
**Left**	20.1 ± 8.3	28.9 ± 10.5	67.8 ± 18.3	1.75 – 39.84	5.97 – 51.72	22.31 – 90.07	5.3*10^-7^	3.6*10^-8^	3.6*10^-8^
**Affected**	21.6 ± 11.7	32.8 ± 14.6	74.8 ± 15.6	5.28 – 71.53	11.68 – 77.75	23.65 – 89.41	1.1*10^-6^	3.6*10^-8^	3.6*10^-8^
**Unaffected**	20.3 ± 8.0	26.1 ± 8.9	66.7 ± 17.9	1.75 – 36.22	5.97 – 51.72	22.31 – 90.45	1.5*10^-5^	3.6*10^-8^	3.6*10^-8^

This table shows the mean ± SD and ranges for the radial extents of CST reconstructions with the three different algorithms used (DTI-based tractography, CSD-based tractography, and MLFT). Discrimination is made between left and right hemispheres as well as tumor-affected and unaffected hemispheres. P-values were computed for the comparisons of radial extents derived from the different algorithms (Wilcoxon signed-rank paired tests with significance level α=0.05). CST, corticospinal tract; SD, standard deviation; DTI, diffusion tensor imaging; CSD, constrained spherical deconvolution; MLFT, multi-level fiber tracking

When comparing radial extents of tumor-affected and unaffected hemispheres, the observed differences are mostly comparable across the algorithms (**Figure 6**). Further, we separately explored the outliers with values above the mean + 2*σ*, given that they were of most interest, while any cases below the mean – 2*σ* were considered in the range of an expected result. As the unaffected hemisphere apparently does not show the same changes in microstructure related to a tumor, the CST extent there should be at least comparable. Patients with considerable radial extent differences (above the 2σ threshold) are all characterized by mass effects extensive enough to cause midline shift and introduce deformations to the bundle in unaffected hemispheres (**Figure 7**), while the opposite is not always true. Depending on the distinct location of the tumor, midline shift may not lead to higher radial extent of the CST in the unaffected hemisphere; for instance, in cases of a more anterior tumor location and midline shift occurrence, no significant alteration was observed in radial extents. Regarding the reasons for the occurred differences, lateral components of the reconstructed CST pathways might be re-oriented as a result of WM compression causing smoothing of the acute angles of the fibers, which allowed the tractography algorithms to reconstruct them. Similarly, the CST pathways in the unaffected hemisphere could have been compressed and deviated in such a way that the angular resolution of the acquisition would not allow to resolve all fiber orientations properly, given that the acquired sequence only included 32 directions by default. In this regard, increasing the order of spherical harmonics used to estimate FODs would also increase the angular resolution, potentially solving the issue (41). Yet, this would require inclusion of a higher number of gradient directions in the sequence (63). At the same time, MLFT already reconstructs pathways closer to the tumor (**Figure 2**), hinting at potentially small tumor-to-CST distances that may exert impact on neurosurgical planning and have implications for patient outcome in terms of motor function and avoidance of surgery-related functional decline.

While the general trajectory and shape of the DTI-based reconstructions tend to be maintained by both MLFT and CSD-based tractography, MLFT improves the coverage of CST reconstructions as provided by DTI-based tractography over those taken from the CSD algorithm (**Figure 8**). Notably, the median coverage of DTI-based CST reconstructions by the CST as delineated with MLFT was higher than 90% for both tumor-affected and unaffected hemispheres. This indicates that the approach does not considerably increase the false-negative rate, while performing better than the CSD algorithm that provides a median coverage below 80%. At the same time, the coverage of the DTI-based CST reconstruction by MLFT as well as CSD-based tractography is inversely correlated to measures for tumor-related mass effects (as estimated by volumes of tumor core and FLAIR-hyperintense zones), which might reflect the effect of tumor-induced WM changes on the estimated orientation distribution by either of the used models (**Table 3**). However, the question arises which method comes closest to the *in-vivo* course and architecture of the CST. The gold standard to test this would be intraoperative DES, which has not been applied to evaluate CSD or MLFT results because of this study’s retrospective design. Yet, there seems high agreement in neurosurgical oncology that techniques should move beyond DTI-based tractography to improve accuracy of tracking results (34–37).

**Table 3:** Correlations for coverage.

	CSD coverage of DTI		MLFT coverage of DTI	
	r	P-value	r	P-value
**Tumor Core Volume**	-0.25	0.12	-0.24	0.14
**Volume of FLAIR-hyperintense zone**	-0.52	<0.01	-0.52	<0.01
**Volume of Tumor Core + FLAIR-hyperintense zone**	-0.48	<0.01	-0.47	<0.01

This table shows the Pearson correlation coefficient (r) and related p-values for the correlations between tumor core volume, volume of FLAIR-hyperintense zone, and volume of tumor core plus FLAIR-hyperintense zone and coverage of the DTI-derived CST for reconstructions using CSD-based tractography or MLFT, respectively (significance level α=0.05). DTI, diffusion tensor imaging; CSD, constrained spherical deconvolution; MLFT, multi-level fiber tracking; FLAIR, fluid attenuated inversion recovery.

One aspect that may further improve tractography using the MLFT algorithm is to combine it with techniques that provide function-based ROIs for seeding. In this regard, previous work has used activation maps derived from functional MRI for ROI placements (64–66). More recently, motor maps derived from navigated transcranial magnetic stimulation (nTMS) have been used for ROI placements (67–72). Of note, it has been demonstrated that nTMS facilitates optimized tracking results for the CST, particularly when the primary motor cortex was in close vicinity of a brain tumor, suggesting that nTMS may be considered the method of choice to achieve proper ROI placements for CST tractography using DTI (71). Comparisons between the three algorithms using nTMS motor maps for seeding may help identify parts of the CST that are underrepresented by DTI- or CSD-based tractography but are evidently connected to the primary motor cortex, which might in particular include fibers with acute angles that could be better delineated with the MLFT method. Additionally, subcortical language network analysis using nTMS-defined ROIs could be part of future work, as to date it has predominantly been performed with DTI-based tractography (68, 73–75).

The main limitation of the MLFT method is related to the accuracy of the estimated FODs in the WM. In a clinical setting neither the number of acquired directions nor b-values are routinely set high, and the accuracy of the fitted diffusion models may therefore be hampered, as the FODs have to be represented by lower-order spherical harmonics. Additionally, the FOD algorithm used does not estimate separate response functions for different tissues (55). An acquisition with multiple diffusion weightings (e.g., multi-shell imaging) would allow to use FOD estimation algorithms that are capable of differentiating multiple tissues (76). Another important limitation of this study is the absence of a correlation of the tractography results derived from CSD and MLFT to findings of intraoperative DES, as it would allow estimation of the sensitivity and specificity rates of these tractography algorithms. This is due to the study’s retrospective character, while conventionally used DTI-based tractography for delineation of the CST has, however, been performed and considered for presurgical planning and intraoperative guidance within the scope of the standard of clinical care.

## Conclusion

The results of this work suggest that tractography of the CST in patients harboring motor-eloquent HGGs may be improved using the proposed MLFT method. This advancement of the CSD principle enabled delineation of the CST with significantly increased radial extent for fibers close to the motor cortex, while maintaining coincidence with DTI-reconstructed CST bundles.

## Data Availability Statement

The data analyzed in this study is subject to the following licenses/restrictions: Analyzed dataset is a subject to medical secrecy and cannot be shared. The derivatives may be provided by the authors upon request. Requests to access these datasets should be directed to Andrey Zhylka, a.zhylka@tue.nl.

## Ethics Statement

The studies involving human participants were reviewed and approved by the Ethikkommission der Technischen Universität München. Written informed consent for participation was not required for this study in accordance with the national legislation and the institutional requirements.

## Author Contributions

Conceptualization was performed by AZ, NS, ADL, AL, and JP. Methodology involved AZ, NS, and ADL. Software involved AZ, FK, AR, and AL. Validation was performed by NS. Formal analysis performed by AZ and NS. Investigation was performed by AZ and NS. Resources provided by JG, BW, BM, SK, CZ, JK, and SS. Data curation was performed by AZ and NS. Writing – original draft preparation was performed by AZ and NS. Writing – review and editing was performed by AZ, NS, FK, AR, ADL, JG, BW, BM, SK, CZ, JK, SS, AL, and JP. Visualization was performed by AZ and NS. Supervision was performed by NS, BM, CZ, JK, SS, AL, and JP. Project administration was performed by NS, CZ, and JK. All authors contributed to the article and approved the submitted version.

## Funding

AZ is supported by the European Union’s Horizon 2020 research and innovation program under the Marie Sklodowska-Curie grant agreement [765148]. BM, BW, and FK are supported through the SFB 824, subproject B12. This project was furthermore supported by the Deutsche Forschungsgemeinschaft (DFG) through TUM International Graduate School of Science and Engineering (IGSSE), GSC 81. The funders were not involved in the study design, collection, analysis, interpretation of data, the writing of this article or the decision to submit it for publication. All authors declare no other competing interests.

## Conflict of Interest

NS received honoraria from Nexstim Plc (Helsinki, Finland) and Philips Healthcare (Best, The Netherlands). SK is consultant for Ulrich medical (Ulm, Germany) and Brainlab AG (Munich, Germany) and received honoraria from Nexstim Plc (Helsinki, Finland), Spineart Deutschland GmbH (Frankfurt, Germany), Medtronic (Meerbusch, Germany) and Carl Zeiss Meditec (Oberkochen, Germany).

The remaining authors declare that the research was conducted in the absence of any commercial or financial relationships that could be construed as a potential conflict of interest.

## Publisher’s Note

All claims expressed in this article are solely those of the authors and do not necessarily represent those of their affiliated organizations, or those of the publisher, the editors and the reviewers. Any product that may be evaluated in this article, or claim that may be made by its manufacturer, is not guaranteed or endorsed by the publisher.

## Abbreviations

3D, three-dimensional; BraTS, Brain Tumor Segmentation; CSD, constrained spherical deconvolution; CST, corticospinal tract; DES, direct electrical stimulation; DTI, diffusion tensor imaging; EOR, extent of resection; FA, fractional anisotropy; FOD, fiber orientation distribution; FLAIR, fluid attenuated inversion recovery; HGG, high-grade glioma; MLFT, multi-level fiber tracking; MNI, Montreal Neurological Institute; MRI, magnetic resonance imaging; PACS, Picture Archiving and Communication System; ROI, region of interest; nTMS, navigated transcranial magnetic stimulation; SD, standard deviation; TFE, turbo field echo; TE, echo time; TR, repetition time; VBG, Virtual Brain Grafting; WM, white matter.
