# Supplementary material for: Tracking the Corticospinal Tract in Patients With High-Grade Glioma: Clinical Evaluation of Multi-Level Fiber Tracking and Comparison to Conventional Deterministic Approaches
Source: Front Oncol. 2021 Dec 14;11:761169. doi: 10.3389/fonc.2021.761169 (PMC8712728; doi:10.3389/fonc.2021.761169)
Supplement: Supplementary file 1 [file DataSheet_1.docx]

Supplementary Material

# Target region from finer brain parcellation

Using FreeSurfer-based multi-scale parcellation (1,2), we have segmented the precentral gyrus subdividing the superior precentral and lateral precentral gyri (Supplementary Figure 1). After that tractography was performed with the same settings as the experiments from the main manuscript. The comparisons of the radial extents for each new target region of each hemisphere are presented in Supplementary Figures 2-5.

MLFT provided reconstructions with a radial extent covering most of the motor cortex in almost all cases. Conversely, DTI- and CSD-based algorithms repeatedly failed to reconstruct pathways reaching the precentral gyri (Supplementary Figures 4, 5, and 7). At the same time, DTI- and CSD-based reconstructions are often comparable to those of MLFT when targeting the superior precentral gyrus (Supplementary Figures 2, 3, and 6). Interestingly, DTI-based tractography reconstructed bundles targeting the superior part of the precentral cortex with higher extent in certain cases as compared to CSD-based reconstructions.

While MLFT achieved superior performance compared to deterministic DTI- and CSD-based reconstructions when using the lateral part of the precentral cortex as the target region, some false-positive pathways may be observed in the MLFT reconstructions (Supplementary Figure 7). Those pathways are primarily branching from pathways that propagate towards the superior precentral part during the first iteration, which is not part of the target region in this case. When using the whole motor cortex as the target region (as presented in the main manuscript), these pathways would not have been used for seeding at the second iteration having reached their destination, thus highlighting the importance of the adequate choice of the target region.


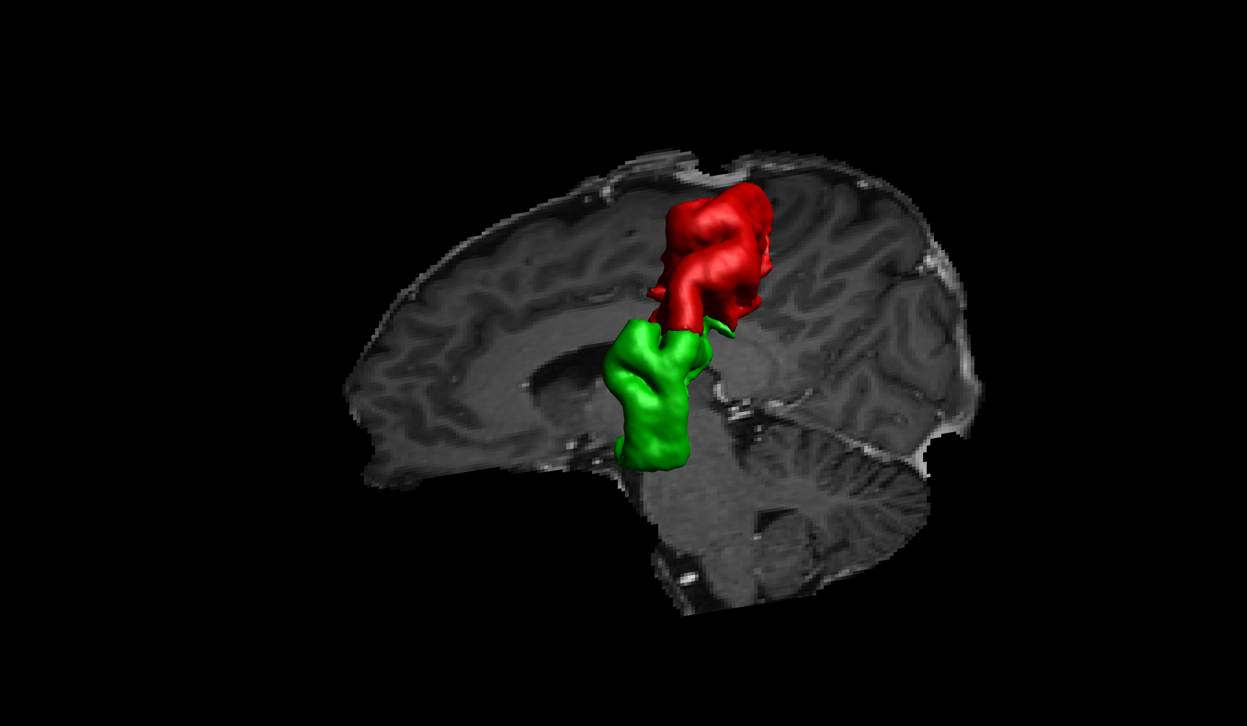


Supplementary Figure 1. Precentral gyrus was subdivided into superior (red) and lateral (green) parts for the experiments exploring the effect of smaller target regions.


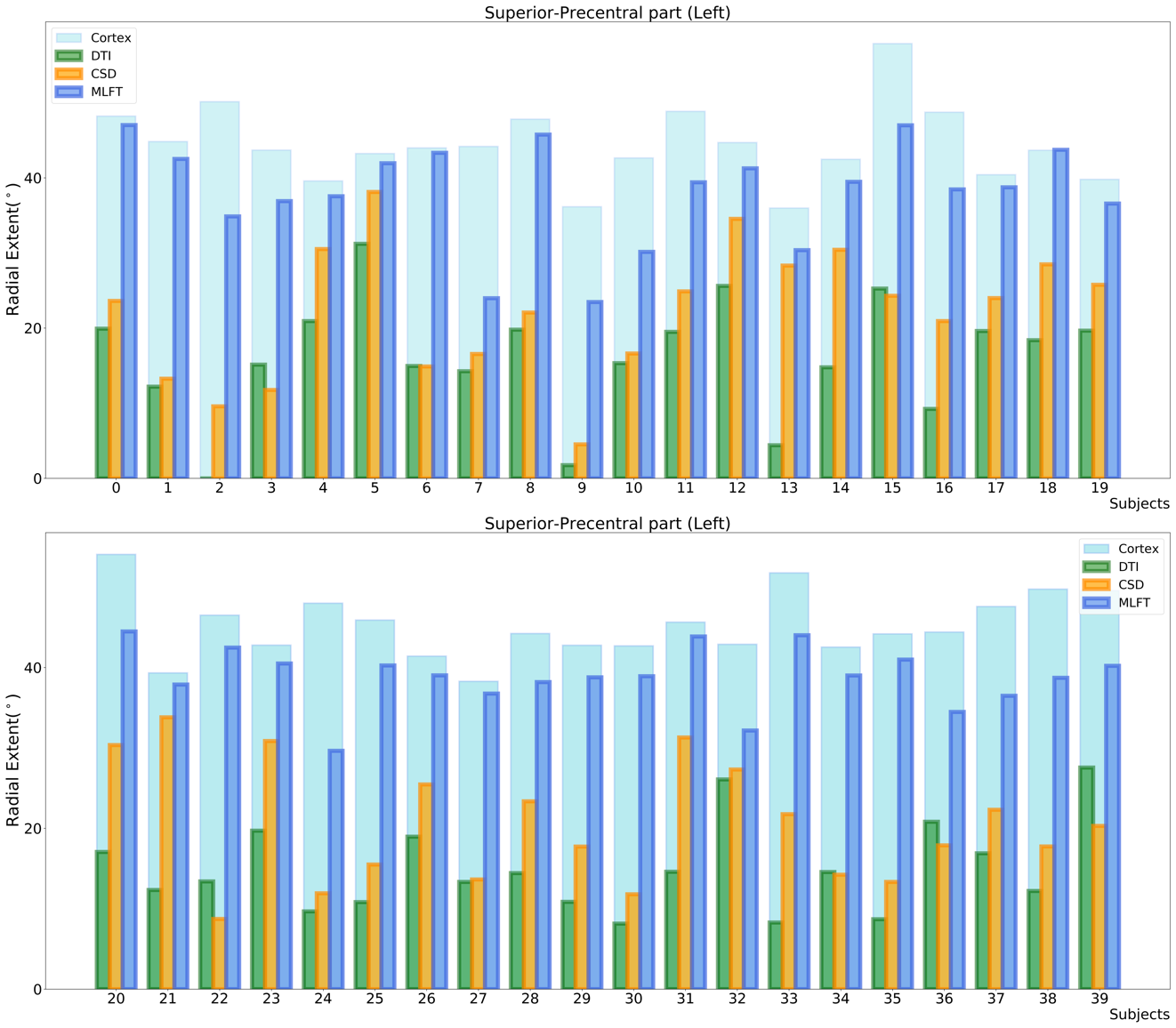


**Supplementary Figure 2.** Comparison of the radial extent of the corticospinal tract (CST) sub-bundle reaching the superior part of the precentral cortex in the left hemisphere. This figure illustrates the radial extent for the reconstructions using diffusion tensor imaging (DTI)-based tractography (green), constrained spherical deconvolution (CSD)-based tractography (orange), and multi-level fiber tracking (MLFT; dark blue). The maximum extent that is the extent of the target cortex part is depicted in light blue.


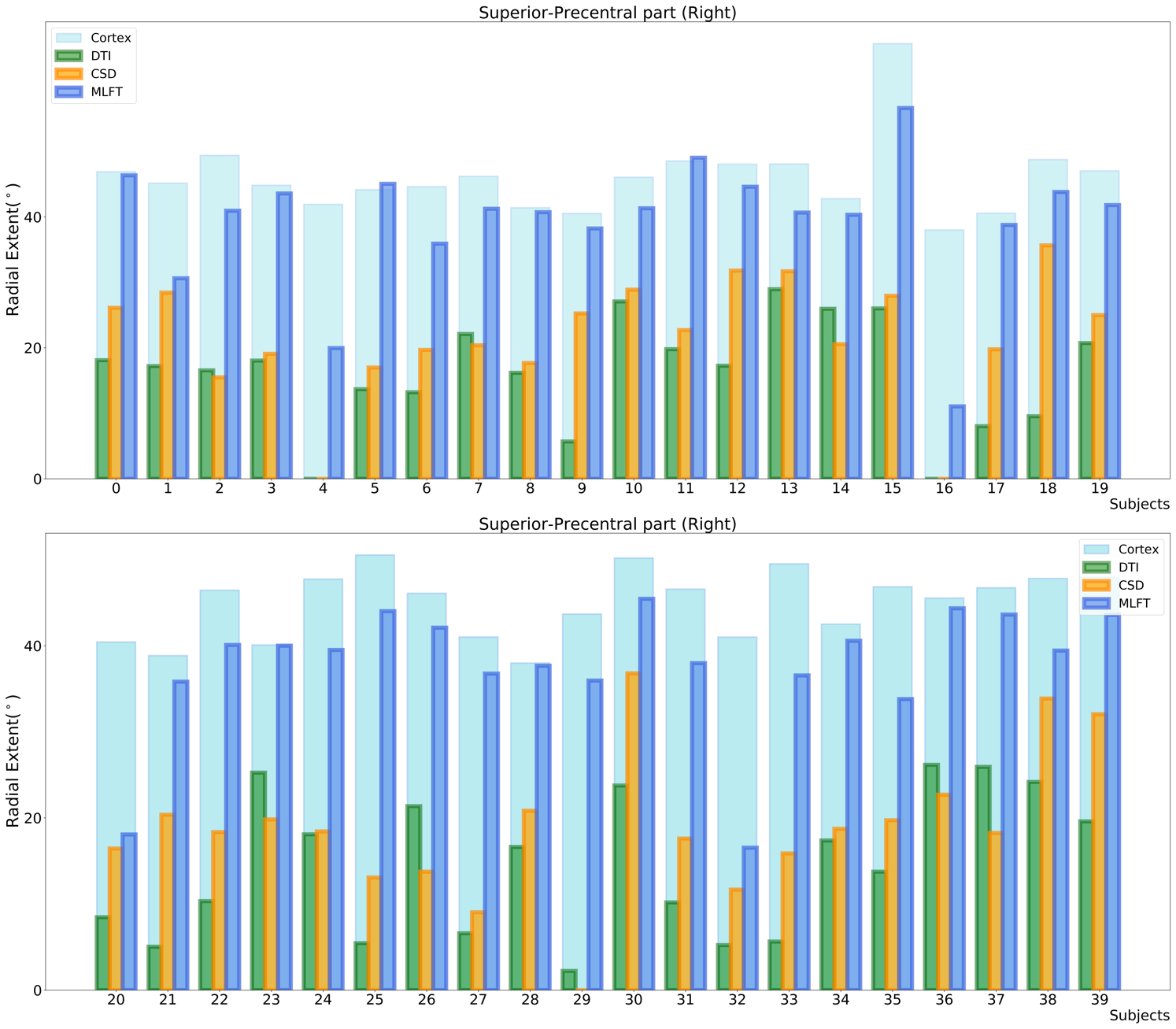


Supplementary Figure 3. Comparison of the radial extent of the corticospinal tract (CST) sub-bundle reaching the superior part of the precentral cortex in the right hemisphere. This figure illustrates the radial extent for the reconstructions using diffusion tensor imaging (DTI)-based tractography (green), constrained spherical deconvolution (CSD)-based tractography (orange), and multi-level fiber tracking (MLFT; dark blue). The maximum extent that is the extent of the target cortex part is depicted in light blue.


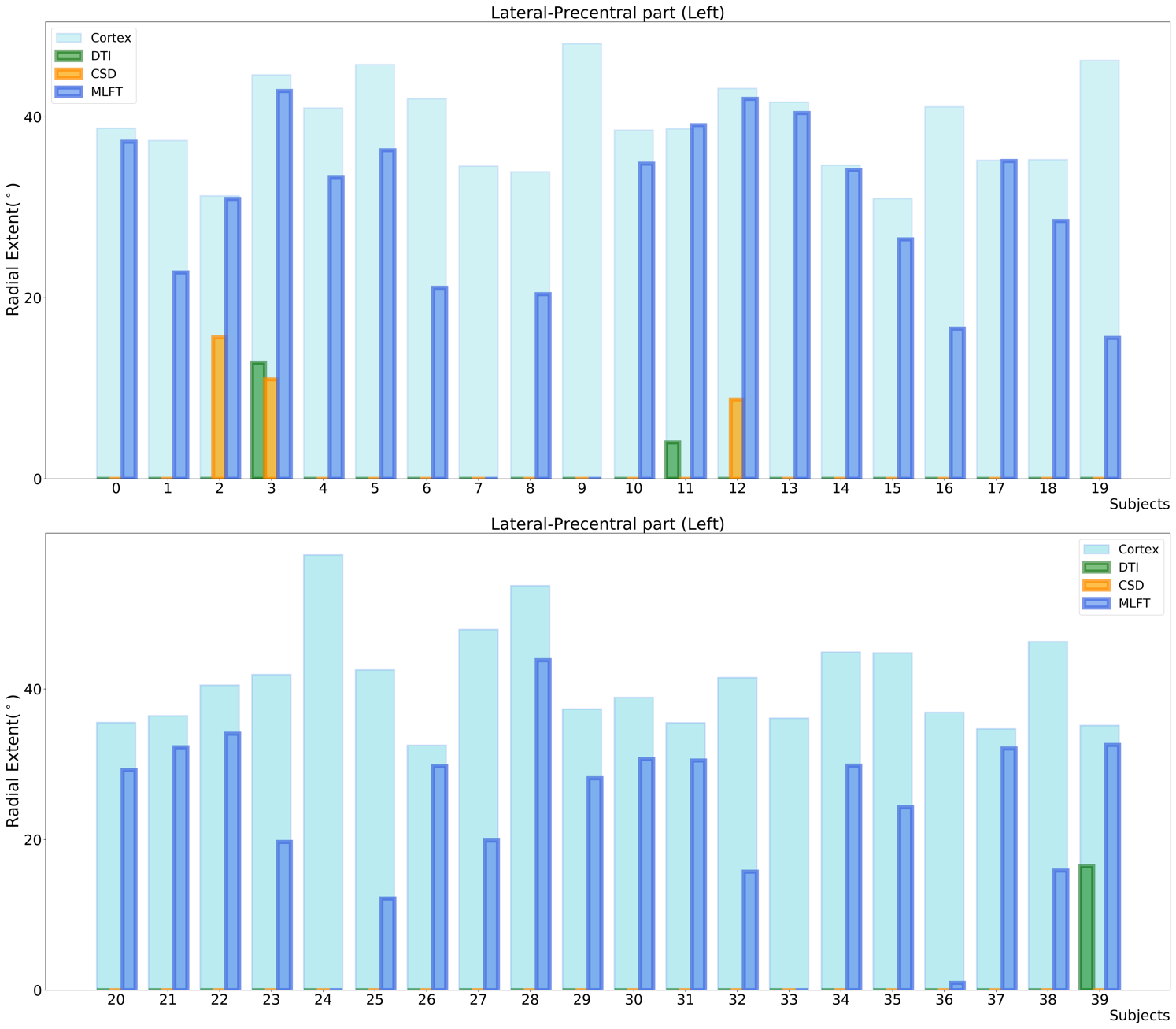


Supplementary Figure 4. Comparison of the radial extent of the corticospinal tract (CST) sub-bundle reaching the lateral part of the precentral cortex in the left hemisphere. This figure illustrates the radial extent for the reconstructions using diffusion tensor imaging (DTI)-based tractography (green), constrained spherical deconvolution (CSD)-based tractography (orange), and multi-level fiber tracking (MLFT; dark blue). The maximum extent that is the extent of the target cortex part is depicted in light blue.


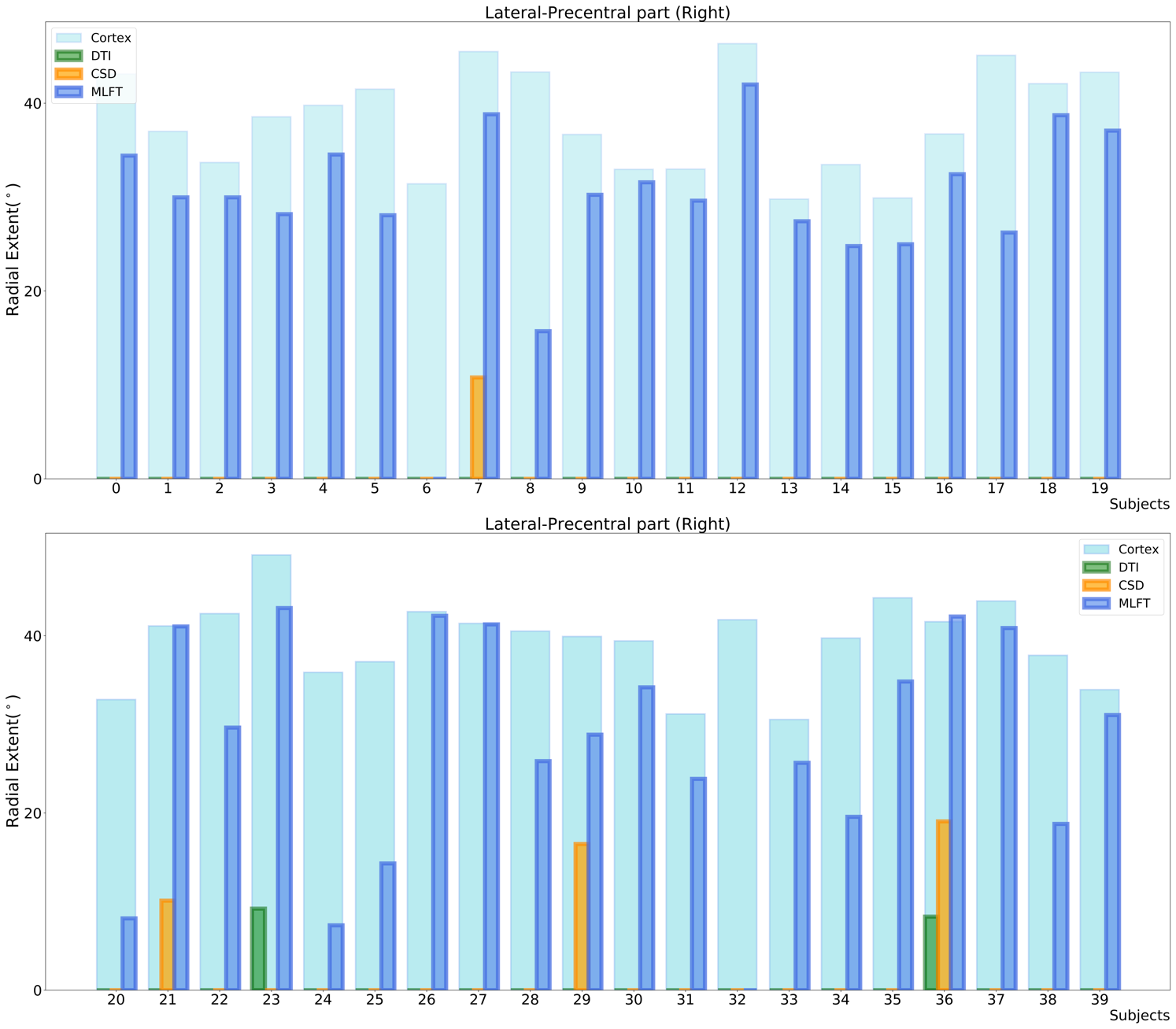


Supplementary Figure 5. Comparison of the radial extent of the corticospinal tract (CST) sub-bundle reaching the lateral part of the precentral cortex in the right hemisphere. This figure illustrates the radial extent for the reconstructions using diffusion tensor imaging (DTI)-based tractography (green), constrained spherical deconvolution (CSD)-based tractography (orange), and multi-level fiber tracking (MLFT; dark blue). The maximum extent that is the extent of the target cortex part is depicted in light blue.


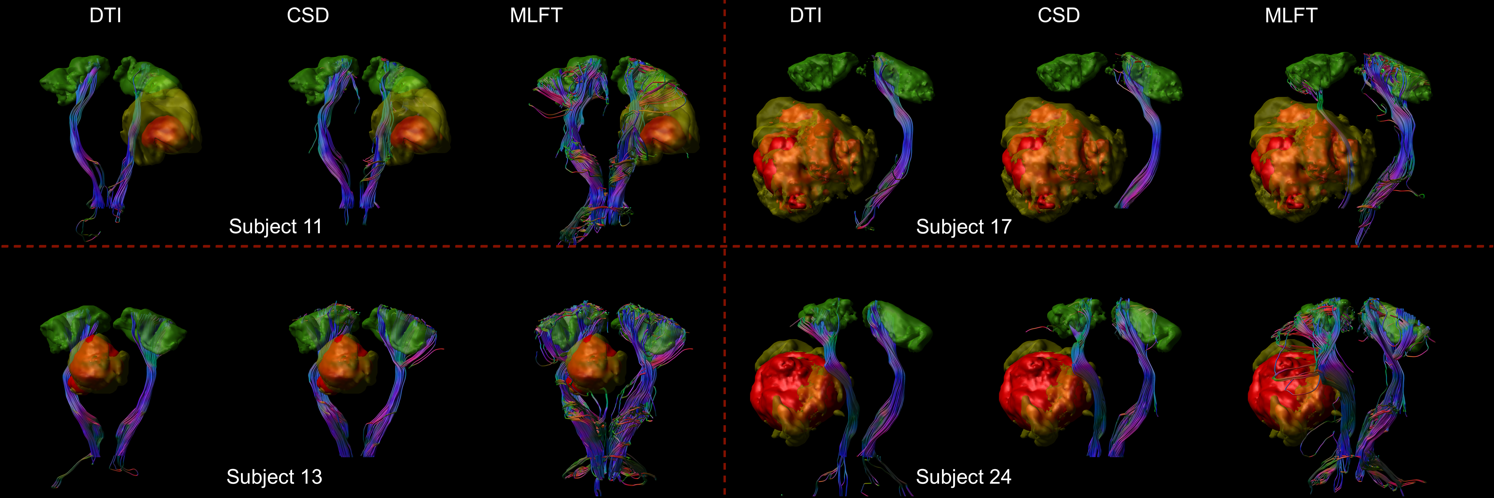


Supplementary Figure 6. Reconstructions of the corticospinal tract (CST) sub-bundles given the superior part of the precentral gyrus (green) as the target region of interest (ROI). In most cases, deterministic diffusion tensor imaging (DTI)-based and constrained spherical deconvolution (CSD)-based reconstructions reach the target region. However, in presence of extensive tumor (red) and edema (yellow), only MLFT was capable of providing a proper fiber reconstruction.


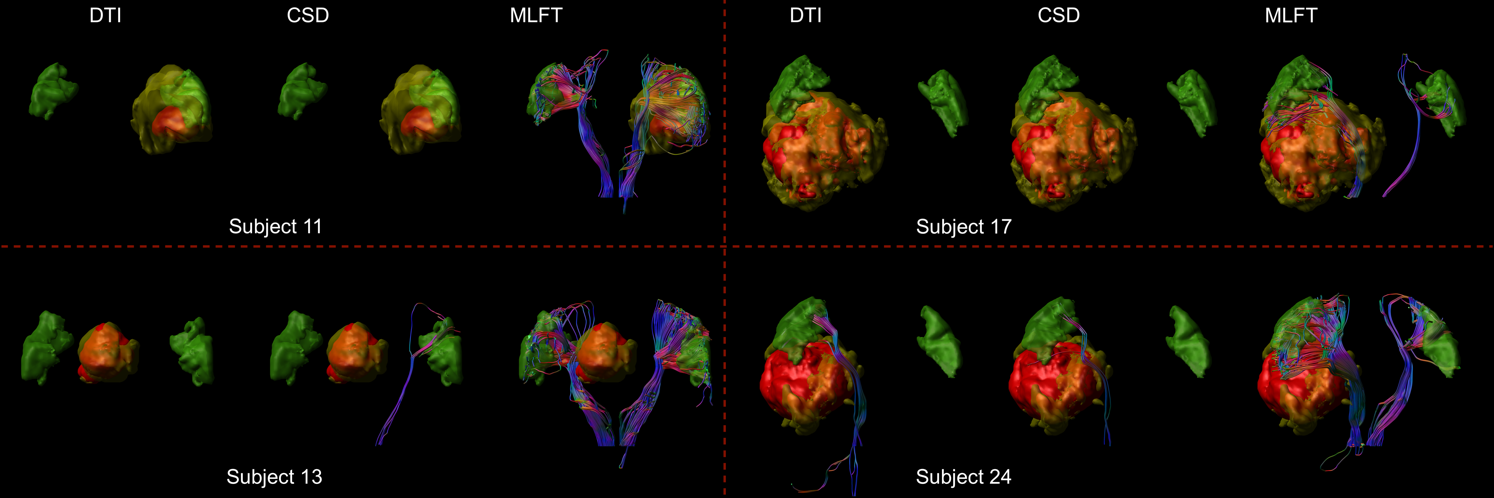


Supplementary Figure 7. Reconstructions of the corticospinal tract (CST) sub-bundles given the lateral part of the precentral gyrus (green) as the target region of interest (ROI). The limitation of diffusion tensor imaging (DTI)-based and constrained spherical deconvolution (CSD)-based algorithms is highlighted as most often these approaches are unable to reconstruct any pathways given such target regions, regardless of tumor size (red: tumor core, yellow: edema).

# References

Tourbier S, Aleman-Gomez Y, Griffa A, Hagmann P (2019, October 22) sebastientourbier/multiscalebrainparcellator: Multi-Scale Brain Parcellator (Version v1.1.0). Zenodo. <http://doi.org/10.5281/zenodo.2536778>

Tourbier S, Aleman-Gomez Y, Griffa A, Bach Cuadra M, Hagmann P (2019, June 12). Multi-Scale Brain Parcellator: a BIDS App for the Lausanne Connectome Parcellation. 25th Annual Meeting of the Organization for Human Brain Mapping (OHBM), [abstract #1714](https://ww5.aievolution.com/hbm1901/index.cfm?do=abs.viewAbs&abs=1714), [poster #W616](https://files.aievolution.com/hbm1901/abstracts/51863/W616_Tourbier.pdf).
